# Supplementary material for: Styrene-Free Bio-Based Thermosetting Resins with Tunable Properties Starting from Vegetable Oils and Terpenes
Source: Polymers (Basel). 2022 Oct 5;14(19):4185. doi: 10.3390/polym14194185 (PMC9572440; doi:10.3390/polym14194185)
Supplement: Supplementary file 1 [file polymers-14-04185-s001.zip › polymers-1931202-supplementary.pdf]

Supporting Information

Styrene-free carbon biobased thermoset resins with tunable properties starting from vegetable oils and terpenes

Fabio Bertini[1,*], Adriano Vignali[1], Marcello Marelli[2], Nicoletta Ravasio[3], Federica Zaccheria[3,*]

[1] Istituto di Scienze e Tecnologie Chimiche "G. Natta" (SCITEC)-CNR, Via Corti 12, 20133 Milano

[2] Istituto di Scienze e Tecnologie Chimiche "G. Natta" (SCITEC)-CNR, Via Fantoli 16/15, 20138 Milano

[3] Istituto di Scienze e Tecnologie Chimiche "G. Natta" (SCITEC)-CNR, Via Golgi 19, 20133 Milano

***** Correspondence: fabio.bertini@scitec.cnr.it (F.B.); federica.zaccheria@scitec.cnr.it (F.Z.)

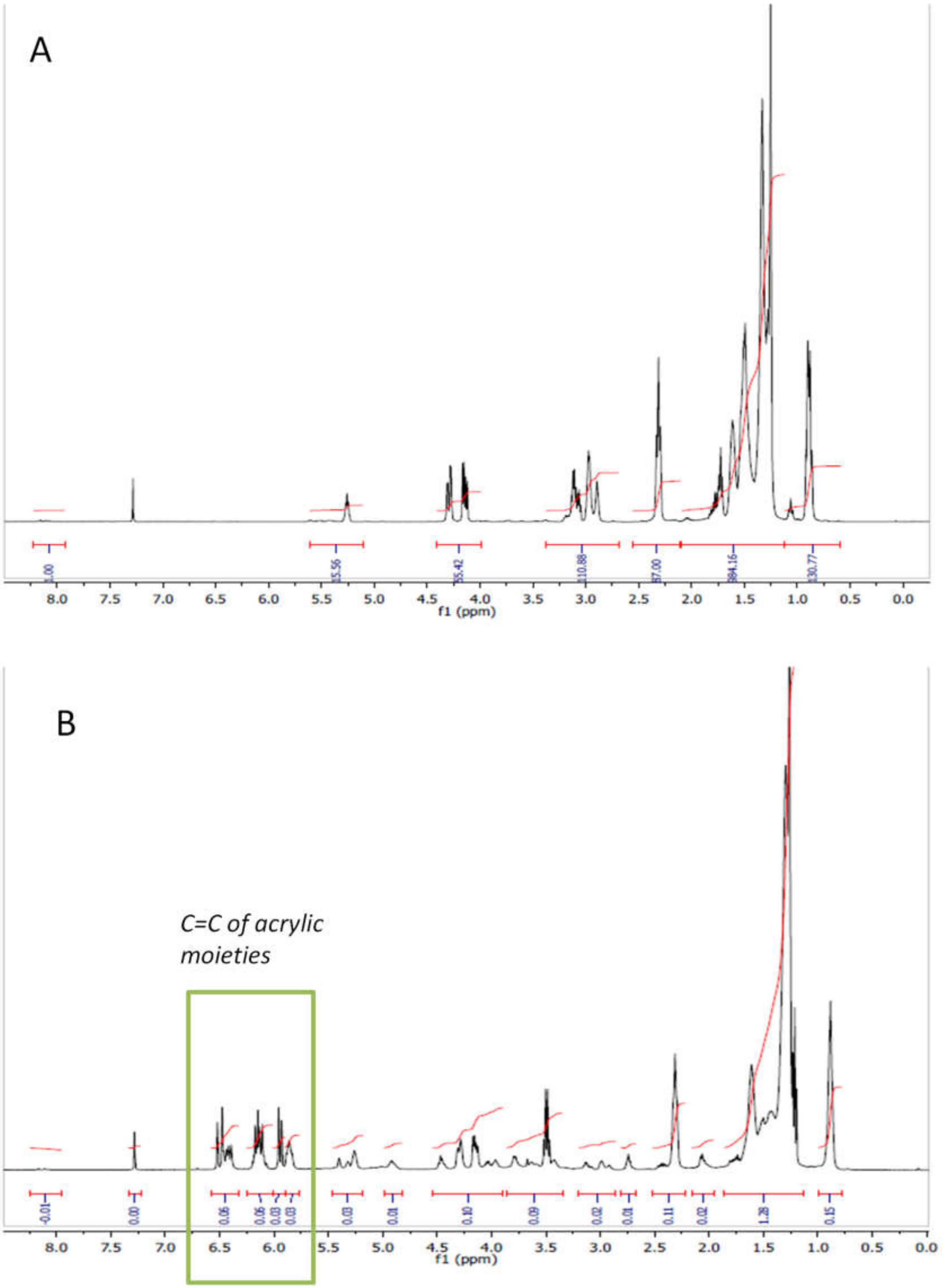

Figure S1. ^{1}HNMR Spectra of A) ESO and B) AESO

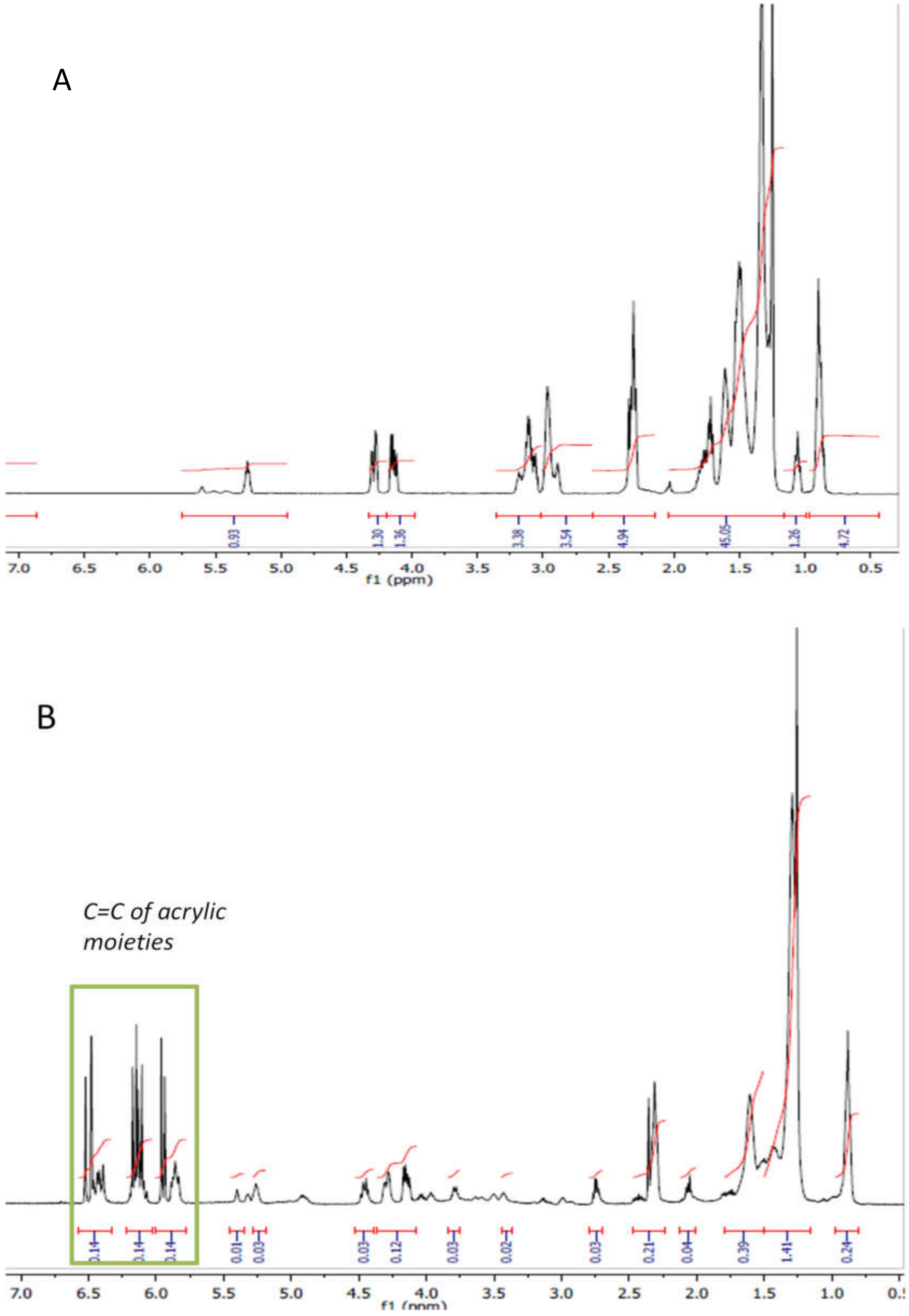

Figure S2. ^{1}HNMR Spectra of A) EHO and B) AEHO

A

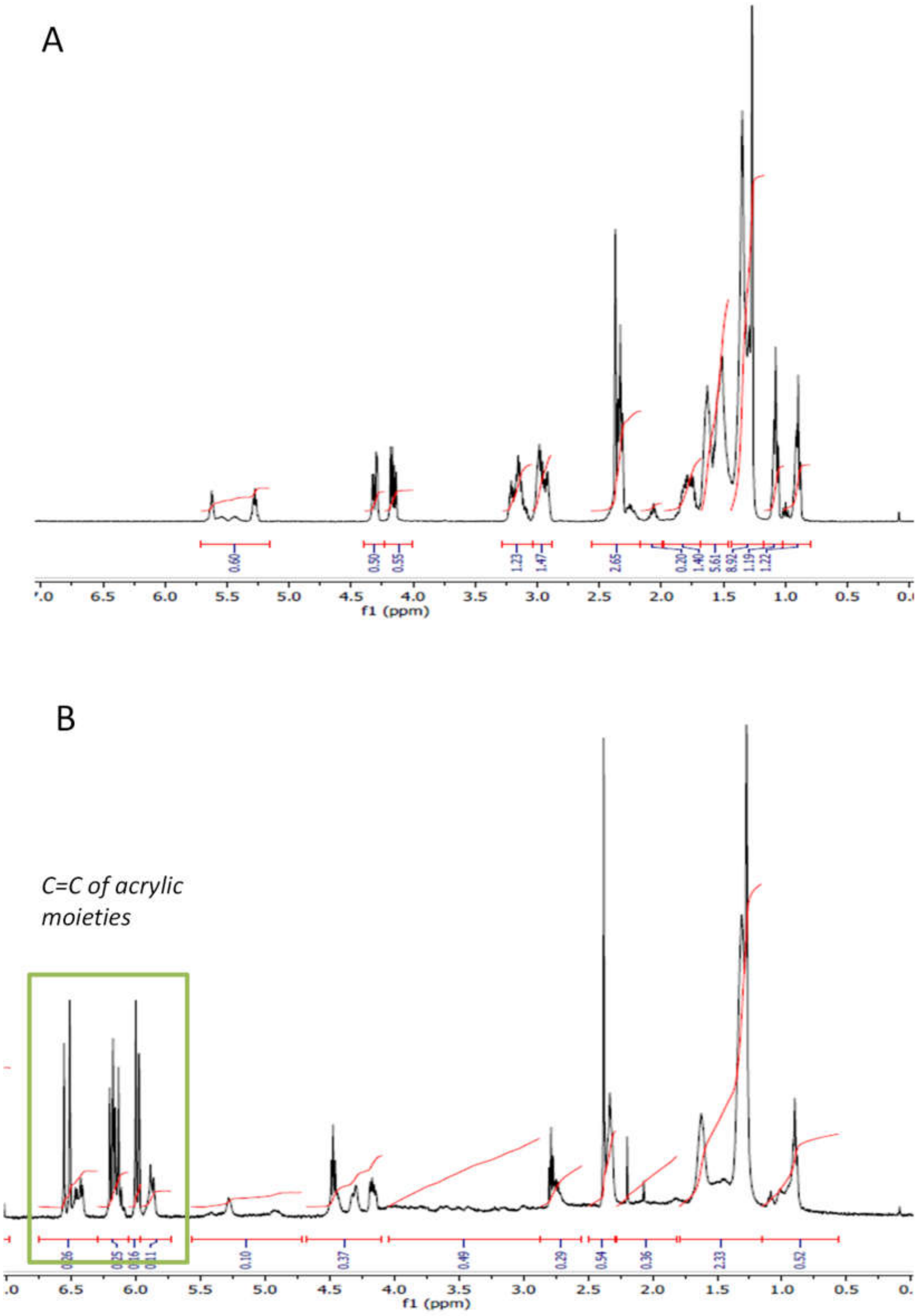

Figure S3. ¹HNMR Spectra of A) ELO and B) AELO

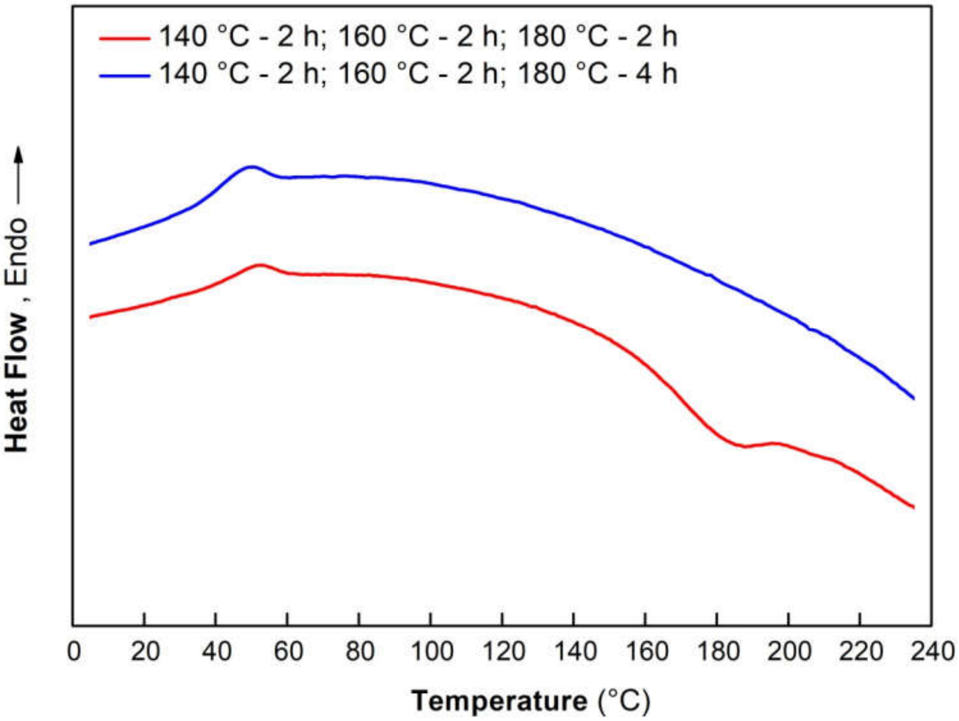

Figure S4. DSC thermograms of AESO-Li resins prepared with preliminary curing (red curve) and optimized curing (blue curve)

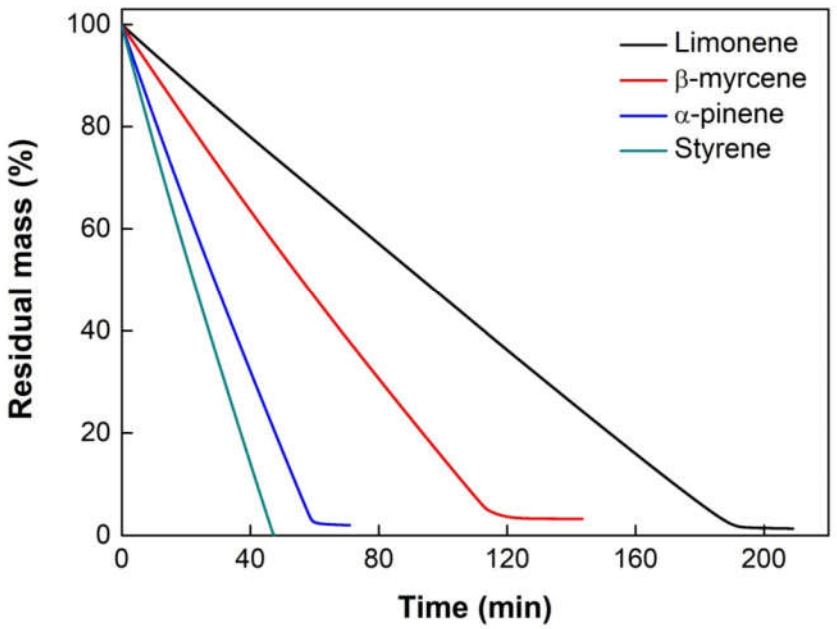

Figure S5. Volatility of co-monomers at 30 °C measured by TGA analysis

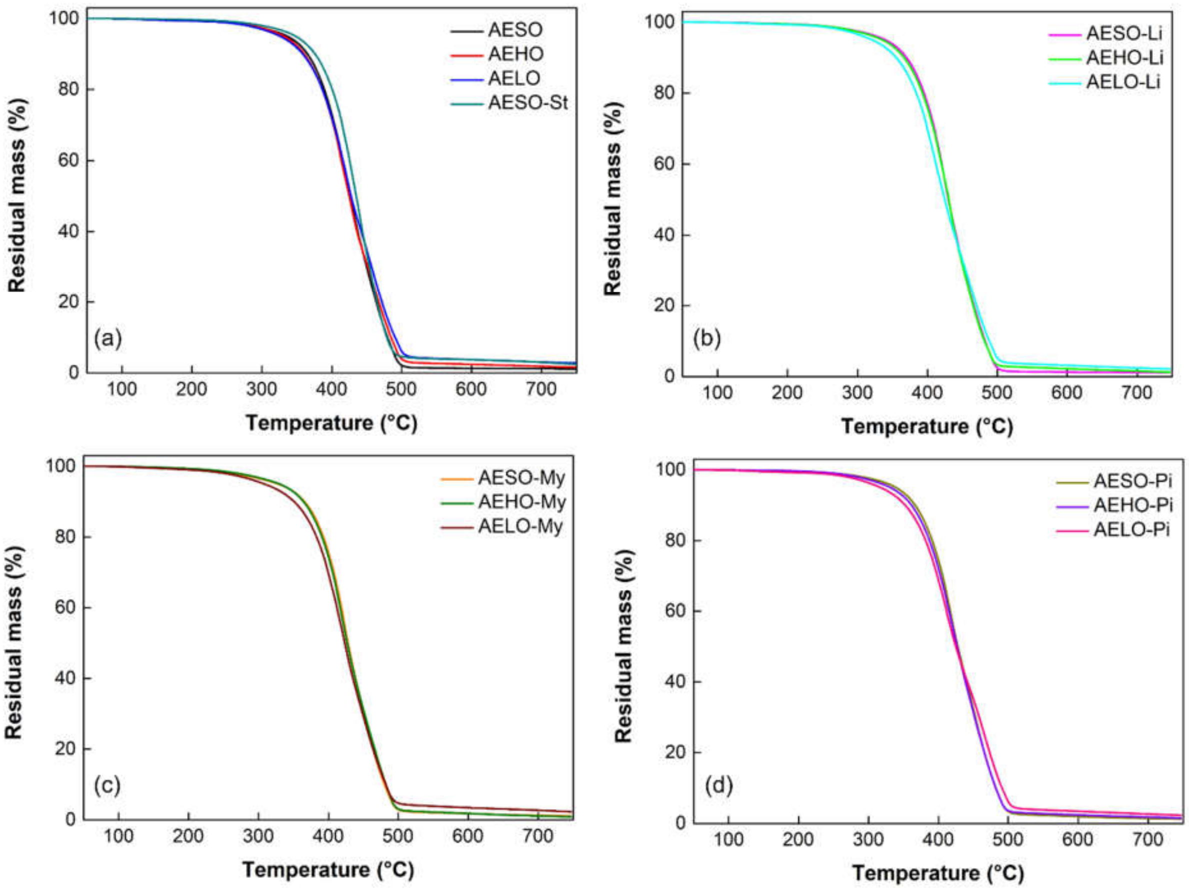

Figure S6. TGA analysis of the resins

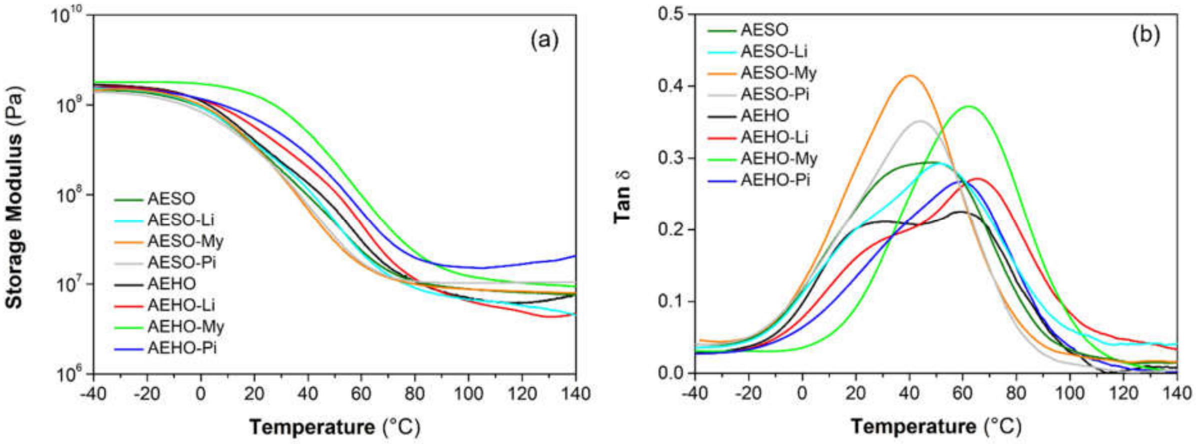

Figure S7. (a) Storage modulus and (b) damping factor as function of temperature for the AESO and AELO based resins

Table S1. Properties of terpenes used as comonomers

**co-monomer**	**b.p. (°C)**	**d (g/cm^3)**
Limonene	177	0.840
β-Myrcene	166-168	0.794
α-Pinene	155	0.86

Table S2. Tensile mechanical properties of resins

**sample**	**E^a (MPa)**	**$\sigma_{max}{}^b$ (MPa)**	**$\varepsilon_r{}^c$ (%)**
**AESO**	258 ± 44	7.4 ± 1.1	5.3 ± 0.1
**AESO-Li**	593 ± 23	12.4 ± 0.2	3.9 ± 0.4
**AESO -My**	680 ± 79	18.7 ± 3.0	4.8 ± 1.4
**AESO-Pi**	192 ± 49	6.4 ± 1.4	6.4 ± 1.0
**AESO-St**	1160 ± 90	21.3 ± 1.9	4.3 ± 1.1
**AEHO**	798 ± 154	13.0 ± 1.8	3.2 ± 1.1
**AEHO-Li**	944 ± 77	16.5 ± 2.5	2.4 ± 0.3
**AEHO-My**	753 ± 61	18.3 ± 3.4	5.4 ± 1.7
**AEHO-Pi**	675 ± 72	11.4 ± 1.2	2.8 ± 0.3
**AELO**	1800 ± 91	25.1 ± 2.8	1.8 ± 0.1
**AELO-Li**	1840 ± 191	17.7 ± 6.8	1.2 ± 0.4
**AELO-My**	1400 ± 105	22.0 ± 2.6	2.7 ± 0.8
**AELO-Pi**	1560 ± 49	18.9 ± 1.9	1.9 ±0.5

[a] Young's modulus; [b] ultimate tensile strength; [c] elongation at break.
